# Study of Polydiacetylene-Poly (Ethylene Oxide) Electrospun Fibers Used as Biosensors

**DOI:** 10.3390/ma9030202

**Published:** 2016-03-16

**Authors:** A K M Mashud Alam, Janet P. Yapor, Melissa M. Reynolds, Yan Vivian Li

**Affiliations:** 1 Department of Design and Merchandising, Colorado State University, Fort Collins, CO 80523, USA; akmmalam@rams.colostate.edu; 2 Department of Chemistry, Colorado State University, Fort Collins, CO 80523, USA; jpyapor@rams.colostate.edu (J.P.Y.); Melissa.Reynolds@colostate.edu (M.M.R.); 3 School of Biomedical Engineering, Colorado State University, Fort Collins, CO 80523, USA; 4 Department of Chemical and Biological Engineering, Colorado State University, Fort Collins, CO 80523, USA

**Keywords:** polydiacetylene, electrospun fiber, biosensor, color transformation

## Abstract

Polydiacetylene (PDA) is an attractive conjugated material for use in biosensors due to its unique characteristic of undergoing a blue-to-red color change in response to external stimuli. 10,12-Pentacosadiynoic acid (PCDA) and poly (ethylene oxide) (PEO) were used in this study to develop fiber composites via an electrospinning method at various mass ratios of PEO to PCDA, solution concentrations, and injection speeds. The PEO-PDA fibers in blue phase were obtained via photo-polymerization upon UV-light irritation. High mass ratios of PEO to PCDA, low polymer concentrations of spinning solution, and low injection speeds promoted fine fibers with small diameters and smooth surfaces. The colorimetric transition of the fibers was investigated when the fibers were heated at temperatures ranging from 25 °C to 120 °C. A color switch from blue to red in the fibers was observed when the fibers were heated at temperatures greater than 60 °C. The color transition was more sensitive in the fibers made with a low mass ratio of PEO to PCDA due to high fraction of PDA in the fibers. The large diameter fibers also promoted the color switch due to high reflectance area in the fibers. All of the fibers were analyzed using Fourier transform infrared spectroscopy (FT-IR) and differential scanning calorimetry (DSC) and compared before and after the color change occurred. The colorimetric transitional mechanism is proposed to occur due to conformational changes in the PDA macromolecules.

## 1. Introduction

When biomaterials are analyzed using biosensors, biological responses can be converted by the biosensor into measurable signals, providing analytic tools for the compositions, structures, and functions of the biomaterials [1,2]. Ideal biosensors can provide short response time, high precision and accuracy, and painless diagnosis in both *in vitro* and *in vivo* applications including infectious disease monitoring, food safety, environmental monitoring, and military biodefense [3]. One promising application of biosensors is to detect pathogens and bacterial infections in hygiene processes, such as wound care and personal care. Current wound care management of pathogen infection may be time-consuming because it usually requires multiple steps including physical examination, imaging of the wound, and sample testing, sometimes resulting in a delay of treatment [4]. Such a delay could be life-threatening in chronic wound management. Quick and easy pathogen-detecting biosensors are, therefore, greatly needed in wound care [5,6]. Recently, conjugated polymers have gained interest in biosensor development because they exhibit reliable bio-sensing activities, which are usually associated with color transitional behaviors [7,8]. Conjugated polymers, thus, potentially provide easy signal analysis, user friendliness, and painless diagnosis in the biosensor development.

Polydiacetylene (PDA) is one of attractive conjugated polymers with color transitional properties. PDAs can be prepared by catalysts-free photo-polymerization, resulting in a highly pure product [7,8]. The PDAs usually have absorption at 650 nm, exhibiting a blue color [7]. When the blue phase PDAs are exposed to external stimuli such as chemical or mechanical stress, the absorption is switched to 550 nm and the color of PDAs becomes red [7,9]. The blue-to-red transformation is visible to the naked eye, which makes PDAs attractive materials for biosensor applications. The color transition is also observed when biological factors are applied to the PDAs, including microorganisms [10] and proteins [11]. Because the sensitivity of sensing activities is important in the use of biosensors, PDAs in different structures have been studied, such as thin films [12,13,14,15], crystals [16], coatings, and fibers [17,18,19], suggesting that the sensitivity is usually increased with an increase in contact surface area. One promising fabrication method that provides high surface areas for sensor materials is electrospinning. In electrospinning, a spinning fluid containing PDA is placed in a syringe loaded on a syringe pump. When a high voltage power is on, an electrostatic force overcomes the surface tension of the spinning fluid to eject a liquid jet from the tip of the syringe needle. The jet then undergoes a stretching process and is eventually deposited on a collector, forming solidified PDA fibers [17]. Yoon *et al.* [18] prepared electrospun fiber mats of PDAs for detecting volatile organic compounds (VOCs) via the blue-to-red color switch. The similar method was also used to make PDA fibers for detecting tracer HCl gas, suggesting a significant sensitivity of PDA-containing electrospun fibers. The color change of PDA electrospun fibers was compared with thin films prepared from the same solution, and PDA fibers exhibited superior sensitivity over the PDA films [20]. The high surface area to volume ratio of the electrospun fibers provided an increased number of sites that interacted with external factors, resulting in high sensitivity and a shorter response time, which is favorable for biosensor applications [21]. Electrospinning of PDAs without another polymer is challenging because the viscosity of PDA solutions is relatively low [18]. When mixed with another polymer that is able to increase the viscosity of the spinning solution, PDAs can be incorporated into fiber composites via electrospinning. The other polymer serves as a supportive component in the fiber composites and hence is named the matrix polymer. Previously, PDA electrospun fibers have been developed with matrix polymers such as polymethyl methacrylate (PMMA) [17,18], polystyrene (PS) [18], tetraethyl orthosilicate (TEOS) [22], and poly (ethylene oxide) (PEO) [17].

We have developed PDA fiber composites and observed a blue-to-red color switch in the composites when they were immersed in bacterial solution at room temperature. The observation suggested a great potential for the PDA composites to be used as biosensors. However, previous discussion indicates that the color switch may be due to other external factors such as temperature. Although it is known that PDAs exhibit a color switch at high temperature [7], little is known on the relationship between the temperature and the colorimetric transition in a PDA-embedded fiber composite. If the PDA fiber composite is used in a biosensor, it is critical to determine if a colorimetric transition is triggered by the change in temperature or by the presence of bacteria in the environment. This paper presents a study on the development of PEO-PDA fiber composites and their colorimetric transitional properties as a function of temperature, potentially used in the biosensors for wound dressings. In this study, PEO was used as the matrix polymer because it has excellent biocompatibility and is often used in internal applications of food, pharmaceutical and personal care products [23]. PEO was mixed with 10, 12-pentacosadiynoic acid (PCDA) in the electrospinning solution. The PCDA was photo-polymerized upon UV-light irritation resulting in PEO-PDA fibers. Electrospinning parameters including polymer concentration, mass ratio of PEO to PCDA in solution, and injection speed were studied. The PEO-PDA fibers were obtained with diameters ranging from 220 nm to 3.4 µm. Colorimetric transitional properties of the obtained PEO-PDA fibers were evaluated using a spectrophotometer and compared before and after the fibers were exposed to temperatures ranging from 25 °C to 120 °C. The colorimetric transition in response to temperature changes varied in the fibers depending on how the fibers were made. The blue-to-red switch was not observed in the fibers until the temperature was more than 60 °C. The results were significantly meaningful because an optimum temperature where bacterial growth in wounds is most rapid and luxuriant is usually 35–37 °C [24]. It suggested that the PEO-PDA fiber composite could be potentially used in wound dressings at normal body temperature range (35–37 °C), directly for detecting biological change, such as bacterial infection. Thermal and chemical properties of the fibers were also analyzed using differential scanning calorimetry (DSC) and Fourier transform infrared spectroscopy (FT-IR) to gain an understanding of the color transition mechanism of the fibers. The results revealed a conformational change involving C-C bond rotation in the PDA macromolecules after the heat treatments. It is hypothesized that the conformational change of the PDA leads to the color change from blue to red in PEO-PDA fibers.

## 2. Results and Discussion

### 2.1. Preparation of PEO-PDA Fibers

**Figure 1:**
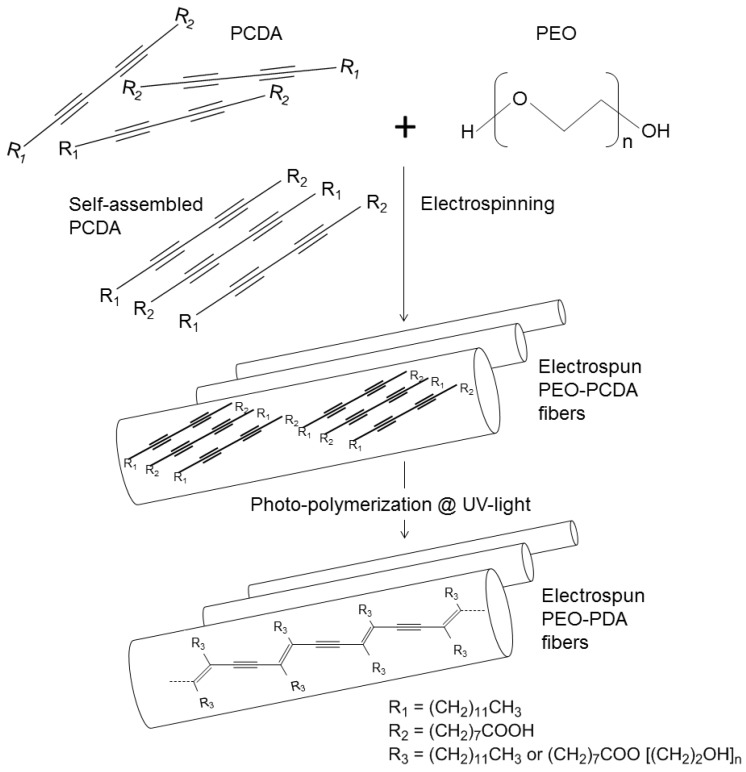
Schematic representation of the electrospinning of poly (ethylene oxide) (PEO) -Polydiacetylene (PDA) composite fibers.

Mixture solutions of PEO and PCDA in chloroform were prepared with varied mass ratios of PEO to PCDA (2, 3, and 4 in Table 1). The solutions were injected by a syringe pump at 0.1 mL·h^−1^ (or 0.2 mL ·h^−1^) and at 15 kV. PEO-PDA fibers in nano and submicron size were obtained on a flat collector placed at a distance of 17 cm. The synthetic scheme of PDA containing fibers is illustrated in Figure 1. When the mixture of PEO and PCDA was stretched during the electrospinning, there were larger attractive forces between the PCDA monomers than those between the PCDA and the PEO matrix, resulting self-assemblies of the PCDA monomers in the obtained fibers. The self-assembled PCDA monomers were then polymerized when the fibers were treated with UV-light irradiation. The fibers were colorless before the UV-light treatment and became blue upon UV-light irradiation, suggesting that the PCDA monomers were polymerized to produce PDAs embedded in the fibers [17].

### 2.2. Characterization of Fiber Morphology

**Figure 2:**
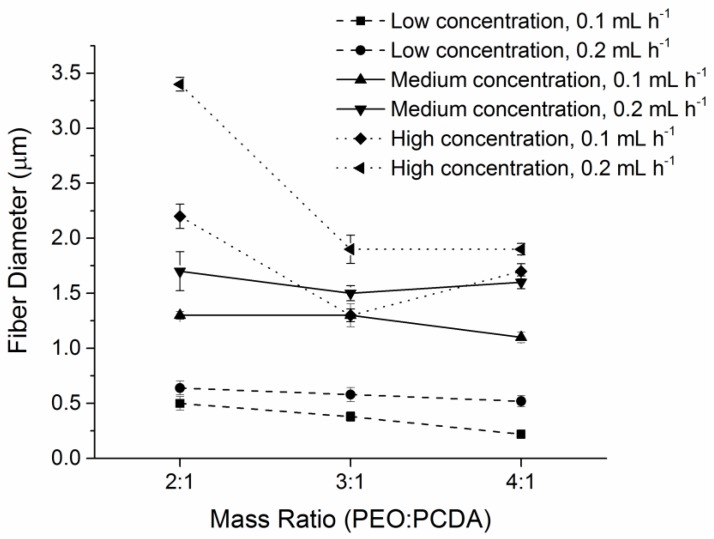
Fiber diameters are illustrated as a function of polymer concentration, mass ratios of PEO and PCDA, and injection speed. The injection speed used was 0.1 mL·h^−1^ (■, ▲ ♦) and 0.2 mL·h^−1^ (●, ▼, ◄), respectively. Finer fibers were formed at higher mass ratios, lower concentrations, and the lower injection speeds.

It was found that the electrospinning parameters of polymer solution concentration, mass ratios of PEO to PCDA, and injection speed had great impact on the morphology of the PEO-PDA fibers. Figure 2 shows average diameters of the fibers prepared at varied parameters using electrospinning. Fine fibers were obtained at high mass ratios of PEO to PCDA, because more PEO in the mixture was able to enhance the fiber formation due to the higher viscosity of PEO supporting fiber stretchability. As expected, the low injection speed (0. 1 mL·h^−1^) produced fine fibers because the amount of polymer solution injected to the spinning region was smaller, resulting finer fibers. Fiber diameters increased with the increase in polymer concentration. The increase in polymer concentration usually prevents rapid changes in the Taylor cone zone of electrospinning, resulting in thick and uniform fibers.

**Figure 3:**
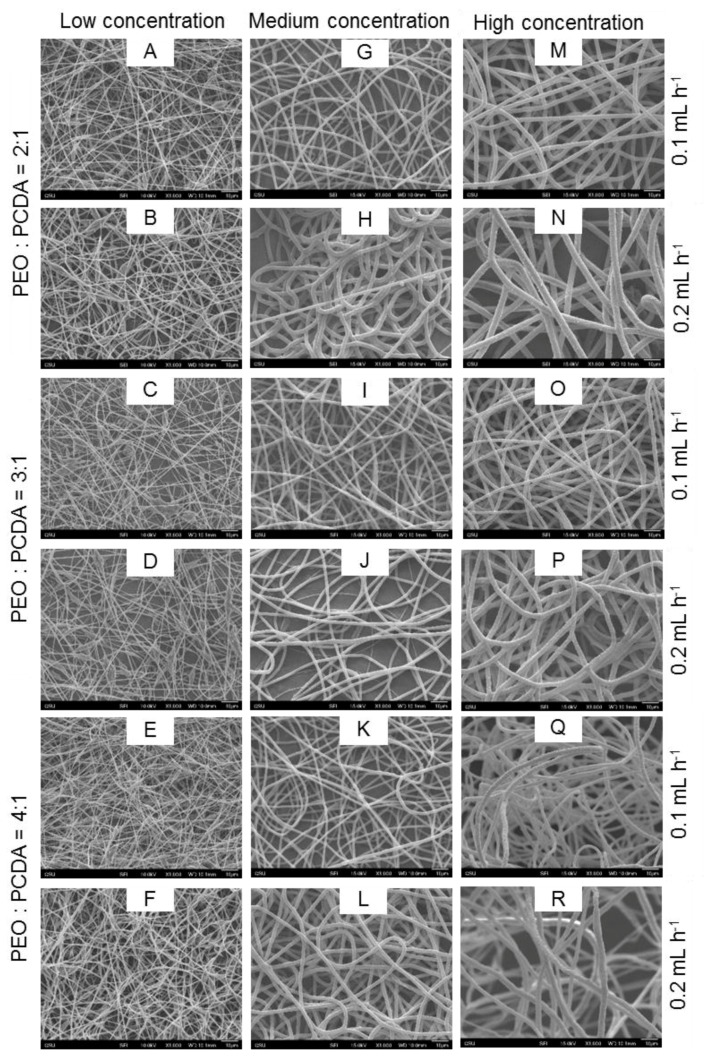
SEM images of PEO-PDA electrospun fibers. Fibers presented in first column (**A**–**F**); second column (**G**–**L**); and third column (**M**–**R**) were prepared at low concentrations, medium concentrations and high concentrations respectively. Respective mass ratio, and injection speed are mentioned respectively at the left and right side of the fiber images. Fibers with beads were prepared at low concentrations (**A**–**F**) irrespective of mass ratio of PEO to PCDA. Number of beads were higher at low (0.1 mL·h^−1^) injection speed (**A**, **C**, and **E**) as compared to the high (0.2 mL·h^−1^) injection speed (**B**, **D** and **F**). Smooth fibers were developed with an increase in concentration and injection speed.

SEM images of the electrospun fibers, shown in Figure 3, illustrate the variation in fiber morphology. The fibers obtained at low concentrations contained beads and junctions (Figure 3A–F). Also, there were more beads in the fibers obtained at a low injection speed (Figure 3A,C,E). The amount of beads on fibers was reduced with an increase of both polymer concentration and injection speed. When the polymer concentration was more than 3 wt. % at any mass ratios, bead-free fibers were formed, indicating a critical concentration of 3 wt. % was required in this study for yielding bead-free fibers. The critical concentration allows polymer chain entanglement that is sufficient for the formation of continuous fibers [25,26]. When the electrically driven polymer jet is elongated in the spinning region, the entanglement of polymer chains prevents them from breaking up, resulting in bead-free fibers.

The surface roughness of the fibers was also varied depending on the electrospinning parameters. High mass ratio of PEO to PCDA promoted fibers with smooth surfaces possibly due to high fraction of PEO in the mixture solution that enhanced fiber formation. It was also found that the surface roughness of the PEO-PDA fibers increased with an increase in solution concentration and injection speed. In previous studies, surface roughness of electrospun fibers containing PDAs was reported to be varied when different solvents were used, including dimethylformamide, chloroform, and methylene chloride [18]. When a mixture of PCDA and poly (methyl methacrylate) (PMMA) in methylene chloride was electrospun, the obtained fibers exhibited porous structures on the surface [17].

### 2.3. Temperature Sensitive Properties of PEO-PDA Fibers

**Figure 4:**
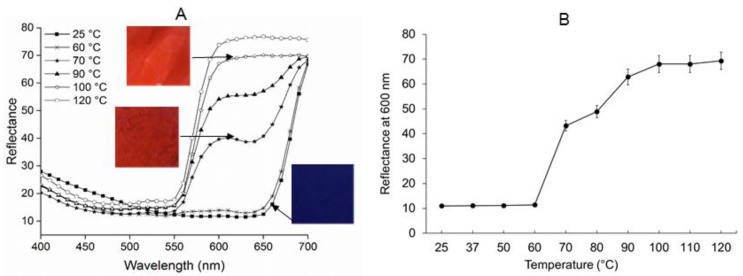
(**A**) Reflectance spectra of the selected PEO-PDA fiber (Fiber #9) treated at selected temperatures (25 °C, 60 °C, 70 °C, 90 °C, 100 °C, and 120 °C). The inserted photographs show the color of the fibers. The fibers treated at 70 °C began to exhibit colorimetric transition on both the reflectance spectra and the photograph. The color switch in the fiber was continuously developed until the temperature was 110 °C. No further change in either reflectance spectra or photograph was observed at 120 °C; (**B**) Reflectance at 600 nm for the fibers treated at different temperature is plotted as a function of temperature ranging from 25 °C to 120 °C. The same color switch behavior was clearly observed when the temperature was more than 60 °C.

Colorimetric transition of the PEO-PDA fibers was evaluated after the fibers were heat-treated at a temperature ranging from 25 °C to 120 °C using a spectrophotometer. 18 PEO-PDA fibers obtained at varied spinning parameters (see Table 1) were all measured using the spectrophotometric method. Representative spectra of fiber #9 are shown in Figure 4. The fiber #9 was obtained at 3:1 mass ratio of PEO to PCDA, injection speed of 0.1 mL·h^−1^, and polymer solution concentration of 3.34 wt.%. The inserted photographs taken at different temperatures are associated with the corresponding reflectance spectra of the fiber.

The fiber exhibited arising and high reflectance at 650 nm to 700 nm at low temperatures (37 °C and 50 °C) and a blue color in the corresponding photograph. When the heat treatment continued for the fiber, the fiber began to demonstrate a reflectance switch to a lower wavelength at 550 nm from 700 nm, resulting in a red color in the corresponding photograph. The reflectance switch was observed in all of the fibers studied in this work, but did not occur until the temperature was more than 60 °C for most of the fibers. It was much significant when the temperature reached 70 °C for all of the fibers (#1–#18). The observation that no blue-to-red switch occurred at low temperatures in the range of normal body temperature could exclude a false positive signal for biological sensing applications, such as bacterial infection.

**Figure 5:**
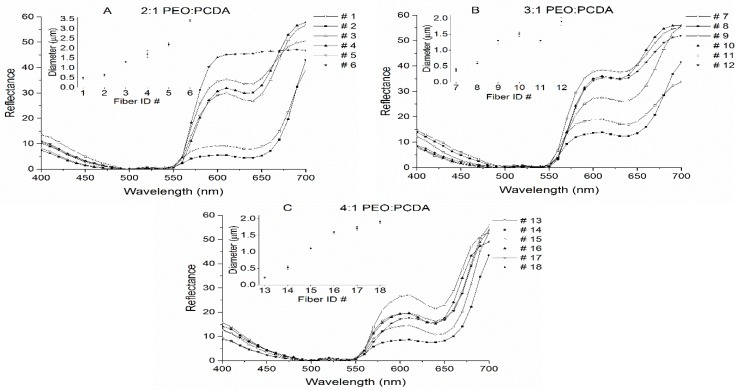
Normalized reflectance spectra of 18 fibers that were heated at 70 °C for 10 min. (**A**) Fibers #1–#6 have mass ratio of PEO to PCDA of 2:1; (**B**): Fibers #7–#12 have mass ratio of PEO to PCDA of 3:1; (**C**): Fibers #13–#18 have mass ratio of PEO to PCDA of 4:1. The inserted figures show the diameters of the corresponding fibers. Fibers #1–#6 exhibit a more pronounced reflectance switch from blue to red.

Normalized reflectance spectra of all the fibers heated at 70 °C are presented in Figure 5 where the diameters of the corresponding fibers are also inserted. The fibers with mass ratio of PEO to PCDA of 2:1 (see Figure 5A) clearly showed a more pronounced reflectance switch (color transition of blue to red) than the fibers with mass ratios of 3:1 and 4:1 (see Figure 5B,C). There was 33.33% PDA in the fiber composite with mass ratio of PEO to PCDA of 2:1. This high fraction of PDA in the fiber composite provided high sensitivity of color switch properties of the fiber. It is also interesting that that the reflectance switch was increasingly pronounced with an increase in fiber diameter, particularly for the fibers with 33.33% PDA (mass ratio of PEO to PCDA of 2:1) (see Figure 5A). Our early discussion on fiber size indicated that the polymer solution and injection speed influenced the fiber size, resulting in an order of fiber diameter as shown in the inserted plots in Figure 5A. The high reflectance associated with the coarse fibers was possibly due to high reflectance surface area. The reflectance switch, or color transition, continuously developed when the temperature was more than 70 °C, but no further change was found after the temperature was more than 110 °C, suggesting that an equilibrium was reached in the PDA macromolecule structure.

**Figure 6:**
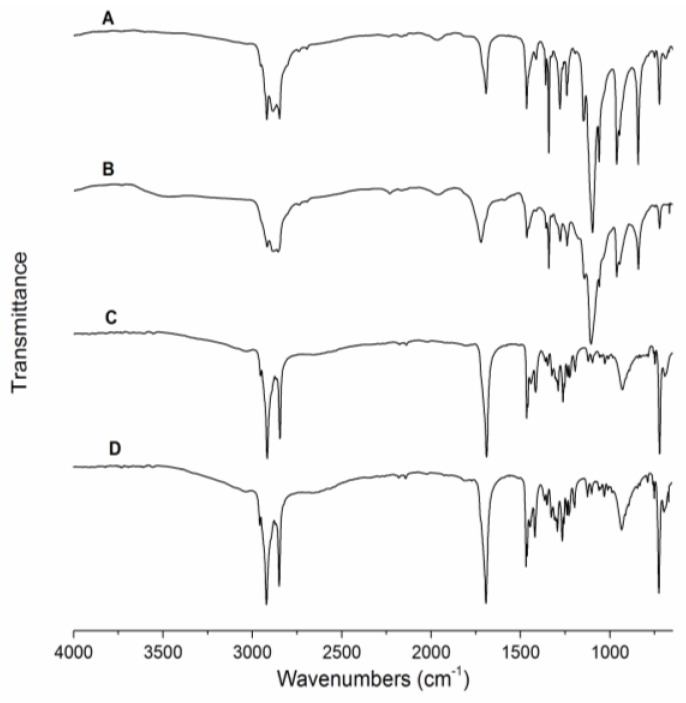
Fourier transform infrared spectroscopy (FT-IR) spectra of PEO-PDA fibers and PDA polymers. The signals correspond to: blue PEO-PDA fibers (**A**), red PEO-PDA fibers (**B**), blue PDA (**C**), and red PDA (**D**).

The blue and red PEO-PDA fibers were compared in FTIR analysis. The results shown in Figure 6 indicate the presence of functional groups expected after the polymerization of PDA in the fibers. Spectrum A corresponds to the blue PEO-PDA fibers, where the following resonance features were interpreted for characterization: IR *ν*_max_ cm^−1^: 2919 cm^−1^ (H-C=C), 2885–2848 cm^−1^ (H-C-H), 1692 cm^−1^ (C=O, ester), and 1097 cm^−1^ (C-O). Similar features were observed in the spectrum B of the red PEO-PDA fibers: IR *ν*_max_ cm^−1^: 2919 cm^−1^ (H-C=C), 2885-2857 cm^−1^ (H-C-H), 1722 cm^−1^ (C=O, ester), and 1107 cm^−1^ (C-O). The resulting resonance features were similar to previously reported results for the PDAs [27,28].

PDA was also synthesized in the absence of PEO and characterized by FTIR before and after the color transition (Figure 6C,D). Spectrum C represents the blue phase PDA where the following resonance features were observed: IR *ν*_max_ cm^−1^: 2955 cm^−1^ (H-C=C), 2918–2847 cm^−1^ (H-C-H), 1690 cm^−1^ (C=O, ester), and 722 cm^−1^ (C=C). Spectroscopic data on spectrum D correspond to the red PDA: IR *ν*_max_ cm^−1^: 2955 cm^−1^ (H-C=C), 2918–2847 cm^−1^ (H-C-H), 1690 cm^−1^ (C=O, ester), and 722 cm^−1^ (C=C).

Previous studies suggested the colorimetric change is due to a twist of the conjugated backbone of the PDAs upon high temperature. The changes reduce the conjugation length and increase the energy band gap in the macromolecular structures of PDAs, which is manifested *via* a color switch from blue to red [7,15]. The similarities found in the IR spectra confirm that the PDA retains its functional groups as it transitions from blue to red. A hypothesis based on these results is that the color change is due to a conformational variation in the side-chains of the PDA which disrupts the π overlap and changes its planarity [29]. This in turn causes changes to the electronic configuration of the PDA, which changes the absorption wavelengths. On spectra A and B, the stretching band associated with the hydrogen-boned carbonyl shifts from 1692 to 1722 cm^−1^, indicating an increase in the C=O bond strength, thus a reduction in the strengh of the hydrogen-bond [30]. This suggests that the C-C rotation creates strain on the polymeric backbone and affects the chemical environment surrounding the alkyne and disfavores hydrogen-bonding on the end groups of the polymer. In addition, the resonance band at 1097 cm^−1^ (A) shifts slightly to 1107 cm^−1^ (B). This subtle change suggests that the matrix polymer (PEO) is also affected by the induced color change. The matrix polymer might be slighly modified as a result of temperature changes which cause oxidation indicated by the resonance band shifts [31]. However, spectra C and D have almost identical vibrational frequencies for the blue and red phases of PDA, where only very subtle changes can be noticed. One such variation is the vibrational frequency at 931 cm^−1^ (C-C), which appears to have higher intensity for the red fibers, suggesting that rotational changes cause the transmittance of the peak to vary. These features and spectroscopic data suggest that changes to the conformation may occur within the backbone of the polymer as it is exposed to increased temperature, disrupting the hydrogen-bonding and causing changes in the electronic configuration of the polymer. The IR results also suggest that the matrix PEO in the PEO-PDA fiber composite does not delay or retard the colorimetric transition in the PDA.

**Figure 7:**
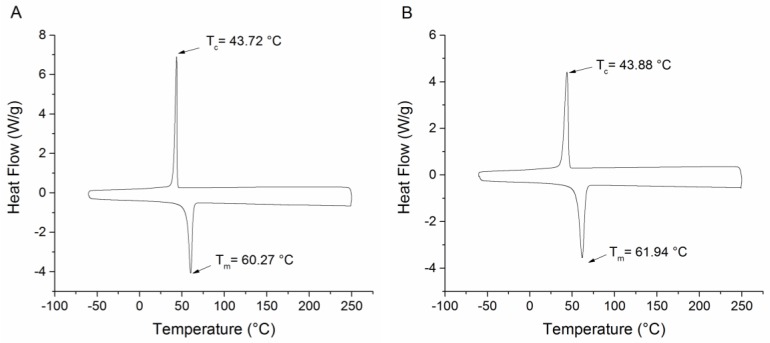
Differential scanning calorimetry (DSC) plot of PEO-PDA fibers: (**A**) blue fibers; and (**B**) red fibers.

Figure 7 illustrates DSC analysis results obtained from the second and third cycles of the blue and red PEO-PDA fibers, respectively. The melting temperature (*T_m_*) and crystallization temperature (*T_c_*) were calculated from the second and third heating cycle, after the thermal history of the mixture of PEO and PDA had been removed. Figure 7A shows the data collected from the blue fibers where *T_c_* was 43.72 °C, and the *T_m_* was 60.27 °C. Similarly, Figure 7B depicts the data from the red fibers where the *T_c_* was 43.88 °C, and the *T_m_* was 61.94 °C.

**Figure 8:**
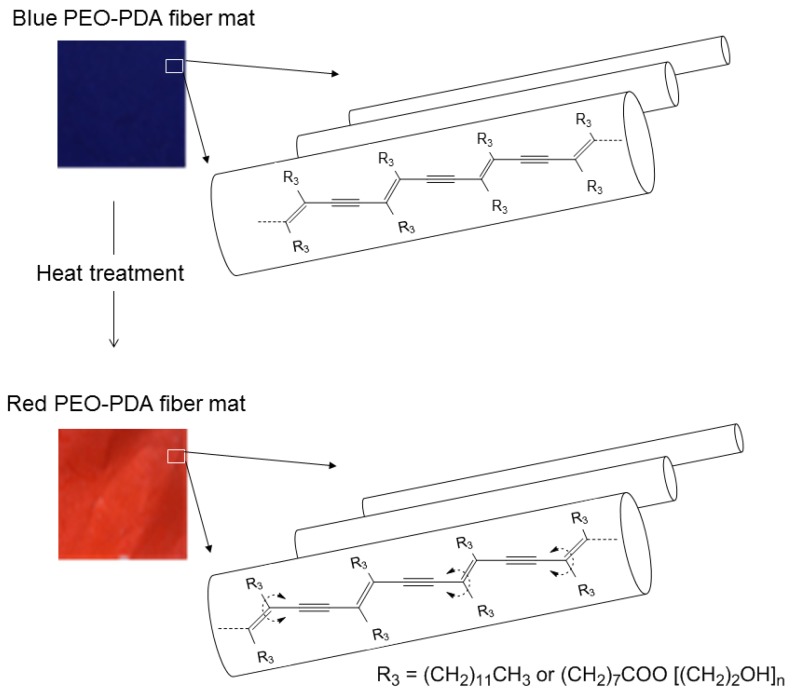
An illustration of color transition from blue to red in PEO-PDA fiber mat, which is due to a C-C bond rotation induced by heat treatment.

Both the blue and red fibers showed similar crystallization and melting temperatures (Figure 7A,B). The crystallization temperatures were determined during the cooling cycle at 43.72 °C and 43.88 °C for the initially blue and red fibers, respectively. This temperature indicates that the crystalline regions in the PEO-PDA fibers became ordered and crystalline, while the amorphous regions provided flexibility to the fibers. It is noteworthy that the melting temperature falls in the range of 60–70 °C, which is the same as when the occurrence of color transition from blue to red is observed in the fibers, as discussed previously. This consistency supports the hypothesis that the optical properties of the material may be influenced by a structural alignment of the polymer chains. As the temperature is increased, C-C bond rotation is facilitated and disruption of hydrogen-bond networks may destabilize the planarity of the polymer confirmation [32]. A change in the conformation of the material is possible due to the phase change of the fibers that increases the entropy of the system, which is shown in Figure 8.

## 3. Materials and Methods

### 3.1. Materials

10, 12-Pentacosadiynoic acid (PCDA, 98%) was the monomer used to prepare polydiacetylene (PDA) and was purchased from GFS Organics (Columbus, OH, USA). Polyethylene oxide (PEO, *M_w_* = 300,000 g/mol). Chloroform (≥ 99.8%) was purchased from Sigma-Aldrich (St. Louis, MO, USA). Diethyl ether was purchased from Fisher Scientific (Fair Lawn, NJ, USA).

### 3.2. Methods

#### 3.2.1. Preparation of PDA

The diacetylene monomer PCDA (6.44 g, 17.2 mmol) was dissolved in diethyl ether (35 mL) and filtered to remove any contaminants. The monomer was isolated after evaporation of the solvent under vacuum in a flask protected from direct exposure to light. Millipore water (18.2 MΩ·cm) was added to yield a 1.29% weight/volume (w/v) suspension, which was sonicated at 65 °C for 30 min. The suspension was allowed to cool to room temperature, then stored at 4 °C overnight. The suspension was transferred to a crystallizing dish with a magnetic stir bar and irradiated with UV light (254 nm) for 8 min [33]. After the photo-polymerization, the dark blue suspension was transferred to a round bottom flask protected from light to remove the solvent under vacuum. The solid PDA was then stored at 4 °C and characterized by FT-IR. ^1^H NMR (Proton nuclear magnetic resonance) characterization was not possible due to the impaired solubility of the material.

#### 3.2.2. Electrospinning of PEO-PDA Fibers

Mixture solutions of PEO and PCDA in chloroform were prepared at different mass ratios of PEO to PCDA (w/w %), different polymer (PEO and PCDA) concentrations, and different injection speed. Table 1 shows the experimental design for preparing PEO-PCDA fibers in the electrospinning.

The PEO and PCDA mixture solutions were stirred overnight on a hotplate stirrer at 350 revolutions per minute (rpm) at room temperature, resulting in a uniform light-pink solution. A customized electrospinning apparatus was used to prepare fibers. The apparatus primarily consisted of a Gamma High Voltage Research ES50P power supply and a Harvard PHD 2000 syringe pump. The uniform PEO-PCDA solution was injected at 0.1 mL·h^−1^ (or 0.2 mL·h^−1^) and 15 KV. The spinning time was kept constant at 1 h, resulting in a thick, colorless fiber mat. The fibers were collected at a distance of 17 cm on a collector plate. The obtained fibers were kept in the dark overnight before UV-light (Spectroline, Longlife^TM^ filter, New York, USA) irradiation. During the irradiation with UV-light at 254 nm, the fibers became blue within 30 s and then turned deep blue in 3 min.

#### 3.2.3. Fiber Characterization

Fiber size and morphology was studied using scanning electron microscope (JEOL, JSM 6500F, Tokyo, Japan). The fiber samples were kept overnight under vacuum to evaporate any residual solvent or moisture. Then they were sputter-coated with gold to improve conductivity of the samples for better quality imaging. FTIR-attenuated total reflectance (ATR) spectra of PDA powders and PEO-PDA fibers were recorded in the range of 650–4000 cm^−1^ using a Nicolet 6700 FTIR spectrometer (Thermo Electron Corporation, Madison, WI, USA). All materials were dried in a vacuum oven overnight at room temperature prior to the analysis. Two spectra were collected for each of the PDA-containing materials, one per color phase. In figure 6, Spectrum A represents the fibers during the blue phase, and B shows resonance features that correspond to the red fibers. The color change was induced by heating the blue fibers at 120 °C for 10 min to obtain red fibers. In addition, spectrum C depicts resonance features of the blue phase of PDAs, and spectrum D corresponds to the red phase of PDAs, where the color change was induced in the same manner as the fibers.

Differential scanning calorimetry (TA Q20 DSC) was used to determine the thermal transitions of the PEO-PDA fibers. The transitions were measured through three heating cycles under nitrogen flow. During the first heating cycle, the temperature was equilibrated at 40 °C and ramped at 10 °C/min to 250 °C and equilibrated at 250 °C. In the second cycle, the temperature was ramped at 10 °C/min to −60 °C and equilibrated at −60 °C. For the third and final cycle, the temperature was ramped at 10 °C/min to 250 °C and equilibrated at 250 °C. Thermolyne benchtop furnace (Thermolyne, Thermo Fisher Scientific, Waltham, MA, USA) was used for exposing the fibers to treatment temperatures up to 120 °C.

#### 3.2.4. Colorimetric Transition Analysis of the PEO-PDA Fibers Due to Temperature Change

The color of PEO-PDA fibers was measured using a spectrophotometer (HunterLab ColorQuest XE). Colorimetric transition behavior of the fibers was evaluated as a function of treatment temperature (25 °C, 50 °C, 60 °C, 70 °C, 80 °C, 90 °C, 100 °C, 110 °C, and 120 °C). Electrospun fiber mat samples (1 inch × 1 inch) were first treated in the benchtop furnace for 10 min at 25 °C. The fiber mat was then measured in the spectrophotometer and reflectance spectra were collected from 400 nm to 700 nm. The same fiber mat was later stored back to the furnace and treated at a higher temperature for another 10 min, following by reflectance measurement of the fiber mat. Reflectance spectra was collected for the same fiber mat after each heat treatment from 25 °C to 120 °C. For a given fiber mat treated at a given temperature, three spectrophotometric measurements were taken and the average reflectance was used for color analysis.

## 4. Conclusions

A mixture solution of PEO and PCDA was used to prepare PEO-PDA fibers via electrospinning. The PCDAs self-assembled in the PEO matrix when the mixture solution was ejected in the electrospinning. The self-assembled PCDAs were photo-polymerized upon UV light irradiation on the electrospun fibers. The size and surface roughness of the fibers was reduced when the mass ratio of PEO to PCDA was increased. A colorimetric change from blue to red was observed when the fibers were treated at a temperature that was higher than 60 °C. The fibers obtained at a mass ratio of PEO to PCDA of 2:1 exhibited pronounced color switch behaviors at 70 °C. No further colorimetric change was found after the temperature was more than 110 °C. High sensitivity of color switch was also associated with low mass ratio of PEO to PCDA in the fibers as well as large size of the fibers. The FTIR and DSC analysis indicated that the color transition was due to a conformational change in PDA macromolecules. The results suggest that the PDA can be embedded into fibers capable of detecting a temperature that is more than 60 °C and signaling this change via a colorimetric change. It is significant that the PDA fiber composite does not change color at normal body temperature (35–37 °C) because this is able to exclude a false positive signal for biological sensing application, such as bacterial infection. No delay or retardation in color switch was observed in the PEO-PDA fiber composite, suggesting that the addition of PEO had no negative impact on the optical properties of the PDA. The study of electrospinning demonstrated that PEO significantly enhanced the spinnability of the PDA. PEO-PDA fiber composites are more economical compared to 100% PDA fiber used in wound dressing, further confirming that it is viable to develop PEO-PDA fiber composites especially for flexible biosensor applications. The responsive behavior of PEO-PDA fiber composites to bacteria is currently undergoing investigation, which will provide more information on the feasibility of using PEO-PDA fiber composites in wound dressings for detecting bacterial infection.

## Figures and Tables

**Table 1:** Electrospinning design for preparing Polyethylene oxide (PEO)-Pentacosadiynoic acid (PCDA) fibers.

PEO:PCDA (w/w)	Polymer Concentration (wt. %)	Injection Speed (mL·h^−1^)	Fiber Identification (ID) Number
2:1	1.5	0.1	#1
		0.2	#2
	3.75	0.1	#3
		0.2	#4
	7.5	0.1	#5
		0.2	#6
3:1	1.33	0.1	#7
		0.2	#8
	3.34	0.1	#9
		0.2	#10
	6.67	0.1	#11
		0.2	#12
4:1	1.25	0.1	#13
		0.2	#14
	3.13	0.1	#15
		0.2	#16
	6.25	0.1	#17
		0.2	#18

## Abbreviations

The following abbreviations are used in this manuscript:

PCDA: 10, 12-Pentacosadiynoic acid
PDA: Polydiacetylene
PEO: Poly(ethylene oxide)
UV: Ultra-violet
SEM: Scanning electron microscope
DSC: Differential scanning calorimetry
FTIR: Fourier transform infrared spectroscopy
NMR: Nuclear magnetic resonance
VOC: Volatile organic compound
PPM: Parts per million
PMMA: Poly(methyl methacrylate)
TEOS: Tetraethyl orthosilicate
PS: Polystyrene
